# Can Obesity Prevalence Explain COVID-19 Indicators (Cases, Mortality, and Recovery)? A Comparative Study in OECD Countries

**DOI:** 10.1155/2022/4320120

**Published:** 2022-06-20

**Authors:** Yuval Arbel, Chaim Fialkoff, Amichai Kerner, Miryam Kerner

**Affiliations:** ^1^Sir Harry Solomon School of Economics and Management, Western Galilee College, Acre 2412101, Israel; ^2^Institute of Urban and Regional Studies, Hebrew University of Jerusalem, Mt. Scopus, Jerusalem 9190501, Israel; ^3^School of Real Estate, Netanya Academic College, 1 University Street, Netanya 4223587, Israel; ^4^Ruth and Bruce Rappaport Faculty of Medicine, Technion, Israel Institute of Technology, Haifa, Israel

## Abstract

SARS-CoV-2 virus disease (COVID-19) is declared a global pandemic with multiple risk factors. Obesity is considered by several researchers as one of the serious risk factors for SARS-CoV-2 virus complications based on recent empirical studies. Yet, other scholars argue in favor of the existence of an obesity survival paradox and criticize the former group of studies on the grounds that they lack controls for race, socioeconomic status, or quality of care. The objective of the current study is to analyze the potential relationships between different SARS-CoV-2 virus indicators and obesity on a country-wide level based on an OECD report. In an attempt to test the counterintuitive possibility of an obesity survival paradox, the proposed empirical model relaxes the assumption of monotonic change by applying the quadratic design and testing which one of the two competing models (i.e., quadratic or linear) better fits the data. Findings suggest more complex relationships between SARS-CoV-2 virus indices and obesity rates than previously thought. Consequently, ethical guidelines referring to priority in intubation and intensive care treatments—published by the Israeli Ministry of Health in April 2020—should account for these complex relationships between obesity and SARS-CoV-2 virus. Indeed, there is a linear increase in mortality rate from SARS-CoV-2 virus with an elevated prevalence of obesity. Yet, other indicators, such as the number of infected per 10,00,000 persons, rates of severe SARS-CoV-2 virus cases, rates of recovered SARS-CoV-2 virus patients, and SARS-CoV-2 virus, as the cause of death exhibit quadratic, rather than linear, patterns. The reasons for these nonlinear patterns might be explained by several conditions such as increased metabolic reserves, more aggressive treatment, other non-SARS-CoV-2 virus complications for obese persons, and unidentified factors that should be examined in future research.

## 1. Introduction

COVID-19 is declared a global pandemic with multiple risk factors (WHO report coronavirus) [1]. Obesity, defined as BMI ≥ 30 where BMI is kg/meter^2^, namely, the weight measured in kilograms divided by the squared height measured in meters (World Health Organization (WHO) : Obesity and Overweight) [2], is considered by several researchers as one of the serious risk factors causing SARS-CoV-2-related complications. SARS-CoV-2 is the virus that causes COVID-19. Simonnet et al. [3] examined 124 COVID-19-hospitalized patients at a medical center in France and found that patients with a body mass index (hereinafter–BMI) > 35 kg/meter^2^ (severe obesity) were 7.36 times more likely to have been put on a mechanical ventilator compared to those with a BMI < 25 kg/meter^2^ (normal weight). According to Garg et al. [4], from March 1 to March 30, nearly half (48.3%) of the patients in COVID-19 from selected US States had obesity (BMI ≥ 30). Yet, other researchers argue in favor of the existence of an obesity survival paradox. Stefan et al. [5] point out that “Conversely, an obesity survival paradox has been observed in patients with pneumonia. That is, despite the increased risk of pneumonia and difficulties of intubation and mask ventilation, the risk of death in patients with obesity and pneumonia might be decreased.”

There are several reasons that may support the former approach, according to which obesity is a risk factor associated with these complications. A summary of a report of more than 72 thousand cases from the Chinese Center for Disease Control and Prevention reported by Wu and McGoogan [6] found that elderly persons (≥ 65 years) and the presence of comorbidities of cardiovascular disease and diabetes mellitus are associated with a more severe course and higher fatality rates of COVID-19 patients. As obesity is strongly associated with an increased risk of cardiovascular disease and diabetes mellitus through direct effects (obesity induced structural and functional adaptions of cardiovascular system to accommodate excess body weight, adipokine effects on inflammation, and vascular homeostasis) and indirect effects (insulin resistance, hyperglycemia, hypertension, and dyslipidemia), a high BMI might be an important risk factor for severe course of COVID-19 disease. According to Kakodkar et al.[7], “COVID-19 clinical presentation can vary from asymptomatic to severe pneumonia” (page 5). As Jones and Nzekwu [8] and Coply et al. [9] demonstrate obesity may restrict the respiratory mechanism of expansion of the lungs, which, in turn, impairs the gas exchange. Indeed, Coply et al. [9] show that reducing obesity after bariatric surgery could improve lung functioning. There are also reasons to believe that obesity may influence the appropriate functioning of the immune system and thus complicate the recovery from COVID-19 (e.g.,—Maurizi et al. [10]). Referring to the association between COVID-19 and obesity, comprehensive reviews are given in de Siqueira et al. [11], Tamara Tahapary [12], and Ovalle et al. [13].

The objective of the current study is to analyze the potential relationships between different SARS-CoV-2 virus indicators and obesity on a country-wide level. The collected indicators are updated to April 28, 2020, and include SARS-CoV-2 virus as the cause of death (of the total number of deaths), number of SARS-CoV-2 virus infections, number of severe cases of illness from SARS-CoV-2 virus, the number of deaths from SARS-CoV-2 virus, and the population of the country as of July 2019. The Organization for Economic Cooperation and Development (OECD) issued a report, which specifies for each country and year the prevalence of overweight and obesity in the population (BMI ≥ 25). The report covers the years 1980–2020. Given the downward bias of self-reported indices, the current analysis uses only the former index.

In an attempt to test the counterintuitive possibility of an obesity survival paradox, the empirical model in the study relaxes the assumption of monotonic change by applying the quadratic design and testing which one of the two competing models (i. e., quadratic or linear) better fits the data. Results of our study confirm the rise in the prevalence of obesity among 23 OECD countries. While in 1980, only 38 percent of the population suffers from overweight and obesity, this percent crosses the 50 percent benchmark (the majority of the population) in 2008 and reaches 55 percent in 2020.

Further results from our study indicate more complex relationships between SARS-CoV-2 virus indices and obesity than previously considered. Differently put, the relationship between COVID-19 indicators and obesity does not necessarily increase or decrease monotonically but rather quadratically. Indeed, there is a linear increase in mortality rate from SARS-CoV-2 virus with the prevalence of obesity. Nevertheless, other indicators, such as the number of infected per 10,00,000 persons, rates of severe SARS-CoV-2 virus cases, rates of recovered SARS-CoV-2 virus patients, and SARS-CoV-2 virus, as the cause of death exhibit quadratic rather than linear patterns.

The remainder of the article is organized as follows: section 2 gives the descriptive statistics; section 3 outlines the methodology, and section 4 reports the results; section 5 provides a discussion of the salient findings; finally, section 6 concludes and summarizes.

## 2. Descriptive Statistics

The data for this article was obtained from the OECD website (available at https://data.oecd.org/). The method is based on regression analysis, the conventional methodology in the empirical literature. For generating all the statistical analyses (data processing, generation of graphs, and statistical analyses), we used Stata software version 16.1. The data set lacks confounders such as population heterogeneity within the various countries.

**Figure 1:**
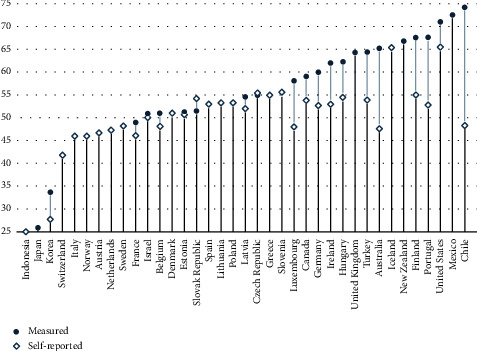
Overweight or obese population (measured/self-reported % of population aged 15+, 2018 or latest available). Source: OECD report available at https://data.oecd.org/pinboard-editor/ (Last accessed on June 30, 2021). Overweight is defined as 25 ≤ BMI < 30, and obesity is defined as BMI ≥ 30, where BMI=kg/meter^2^.

Overweight and obesity are known risk factors for long series of health problems, and the fourth leading risk factor for global mortality [14, 15].  Figure 1 displays the percent of overweight in OECD countries, where BMI = (kg/[(meter)]^2^), overweight is defined as 25 ≤ BMI < 30, and obesity is defined as BMI ≥ 30 (WHO: Obesity and overweight, available at: https://www.who.int/news-room/fact-sheets/detail/obesity-and-overweight). According to the graph, the frequency of overweight stretches from a minimum of 25 percent in Indonesia to a maximum of 74.2 percent in Chile. However, based on measured, rather than self-reported, BMI, Japan and South Korea seem to exhibit the lowest frequency of overweight and obesity (25.65 percent and 34.1 percent, respectively), and Mexico, USA, and Chile exhibit the highest frequencies (71 percent, 72.5 percent, and 74.2 percent, respectively).

**Figure 2:**
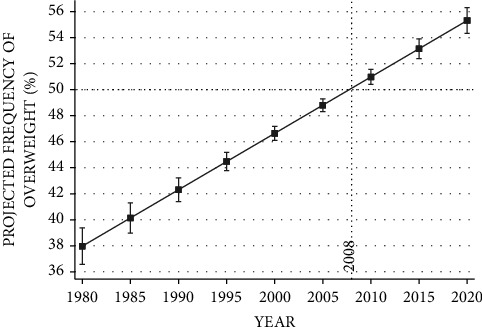
Changes in overweight and obesity prevalence in 23 OCED countries, 1980–2020. The vertical axis measures the projected relative frequency of overweight and obesity (BMI ≥ 25 where BMI=kg/meter^2^) in the population of 23 OECD countries based on measured height and weight. The horizontal axis measures the year in which the measurement took place. The figure is based on the following fixed-effect regression outcomes given in column (2).

Figure 2 exhibits changes in overweight and obesity prevalence among 23 OECD countries between 1980 and 2020, based on measured BMI. Results show that the YEAR variable explains 60.9% of the dependent variable's variance. The baseline projected frequency in 1980 is 37.97 percent. This projected frequency steadily rises by 0.434 annually, crosses the 50 percent benchmark in 2008, and reaches the peak of 55.33 percent in 2020.

**Table:** 

Variables	(1)	(2)
	Overweight	Overweight
(Year 1980)^2^	−0.00392^∗^	—
	(0.0960)	—
(Year 1980)	0.589^∗∗∗^	0.434^∗∗∗^
	(9.00*e* − 09)	(<0.0001)
Constant	36.91^∗∗∗^	37.97^∗∗∗^
	(<0.0001)	(<0.0001)
Observations	180	180
Countries	23	23
*R*-squared	0.616	0.609

*P* values are given in parentheses. ^∗^*P*<0.1; ^∗∗^*P*<0.05; ^∗∗∗^*P*<0.01

To permit appropriate cross-country comparisons, all the variables were transformed to percent according to population size and number of COVID-19 cases.  Table 1 defines the variables by percent. Based on these definitions, Table 2 reports the descriptive statistics of these variables on a country-wide level.  Table 3 provides the descriptive statistics of the pooled sample and gives the medians, means, standard errors, and 95%, 99% confidence intervals of the means.

The respective median and average number of infected people per 10,00,000 persons are 243 and 1,276, respectively, and the standard error is 153 (INFECTED). The implication is right-tailed distribution of INFECTED, namely, relatively few countries with large number of infected people per 10,00,000 persons. The relative frequency of severe cases divided by total COVID-19 cases is 1.25 percent, the median is 0.99, and the standard error is 0.0577 percent (SEVERE). Referring to the severity of outcomes, the reason the authors do not provide an exact definition of severe cases is the complexity of such a definition in different countries, as part of personal characteristics including background diseases, and in contrast to COVID-19 cases (defined by the PCR outcomes) and mortality (as clearly defined by the medical profession. The relative frequency of recovered divided by total COVID-19 cases is 46.57 percent, the median is 38.15, and the standard error is 2.44 percent (RECOVERED). The relative frequency of SARS-CoV-2 virus as the cause of death divided by the total number of dead persons in the country is 8.73 percent, the median is 1.5, and the standard deviation is 0.815 percent (COVID-19). The relative frequency of mortality from SARS-CoV-2 virus divided by the total number of SARS-CoV-2 virus cases in the country is 5.15 percent, the median is 2.83 percent, and the standard error is 0.33 percent (MORTALITY). The 95% and 99% confidence intervals of all the SARS-CoV-2 virus indicators demonstrate that the sample means of all the variables across these 23 countries are different from zero. A mean-median comparison indicates a right-tailed distribution of all these variables, namely, relatively few countries with higher prevalence. Finally, the relative frequency of overweight across these 23 countries and based on 1980–2020 measured BMI is 48.49 percent, and the median is 54.6 percent. The standard error is 1.26 percent (OVERWEIGHT). Based on the confidence intervals, one cannot reject the null hypothesis that the relative frequency equals the 50 percent benchmark, namely, a majority of the population suffers from obesity. Finally, this distribution is left-tailed, namely, there are only few countries with lower prevalence of overweight. This provides evidence regarding the extent of the obesity pandemic in OECD countries.

## 3. Methodology

Consider the following two competing empirical models:

(1) $$\begin{array}{c} Y_{j}=\alpha_{1j}{\text{OVERWEIGHT}}^{2}+\alpha_{2j}\text{OVERWEIGHT}+\alpha_{3j}+u_{1j}, \end{array}$$

(2) $$\begin{array}{c} Y_{j}=\beta_{1j}\text{OVERWEIGHT}+\beta_{2j}+u_{2j}, \end{array}$$

where *j* = 1, 2, 3, 4, 5; *Y*_1_=INFECTED=1,000,000 · (COVID19_CASES/POPULATION); *Y*_2_=SEVERE=100 · (SEVERE_CASES/COVID19_CASES); *Y*_3_=RECOVERED=RECOVERY_CASES/COVID19_CASES; *Y*4=COVID-19 (as the cause of death from the total number of dead persons); Y_5_=MORTALITY=100n(DEATH_CASES/COVID19_CASES); *α*_1*j*_, *α*_2*j*_, *α*_3*j*_ and *β*_1*j*_, *β*_2*j*_ are parameters; and *u*_1*j*_, *u*_2*j*_ are the stochastic random disturbance terms. Unlike equation (2), equation (1) permits nonmonotonic change of the SARS-CoV-2 virus indicator with the OVERWEIGHT variable.

To test which of these two models better fits the data for each indicator, the Ramsey RESET test is employed (e.g., [16]: 270–271). The test is based on estimating the models: $Y_{j}=a_{j}+b_{j}X+c_{j}{\hat{Y}}_{j}^{2}+\mu_{j}$ where ${\hat{Y}}_{j}={\hat{d}}_{j}+{\hat{e}}_{j}X$; *j* = 1,2,3,4,5; *X* = OVERWEIGHT; H0: *c*_*j*_=0; and H1: *c*_*j*_ ≠ 0. Rejection of the null hypothesis suggests that compared to the linear model, the quadratic model fits the data better.

## 4. Results

**Figure 3:**
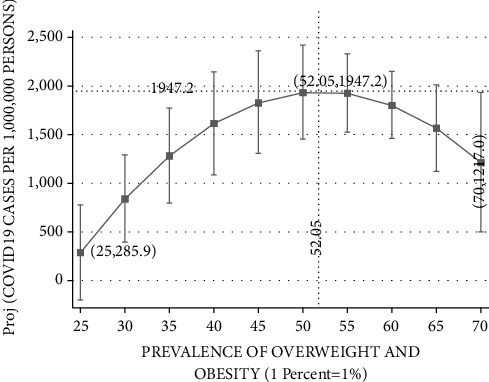
COVID-19 cases per 10,00,000 persons vs. prevalence of overweight and obesity. The vertical axis measures the projected probability of COVID-19 cases per 10,00,000 persons based on the formula: 1,000,000(COVID19_CASES ÷ POPULATION) applied separately to 23 OECD countries. The horizontal axis measures the frequency of overweight and obesity (BMI ≥ 25 where BMI=kg/meter^2^) in the population of OECD countries based on measured height and weight. The figure is based on the following regression outcomes given in column (1).

**Figure 4:**
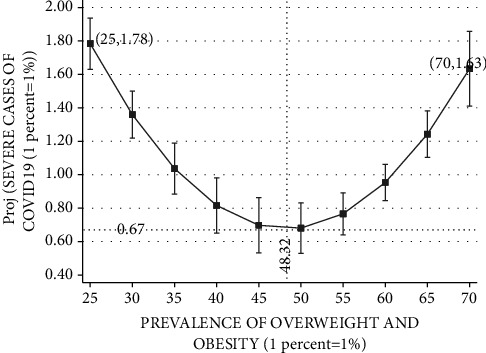
Rate of severe COVID-19 cases vs. prevalence of overweight and obesity. The vertical axis measures the projected probability of severe cases of COVID-19 divided by total cases of COVID-19 in percentage points. The horizontal axis measures the prevalence of overweight and obesity (BMI ≥ 25, where BMI=kg/meter^2^) in the population of OECD countries based on measured (rather than self-reported) height and weight. The figure is based on the following regression outcomes given in column (1).

**Figure 5:**
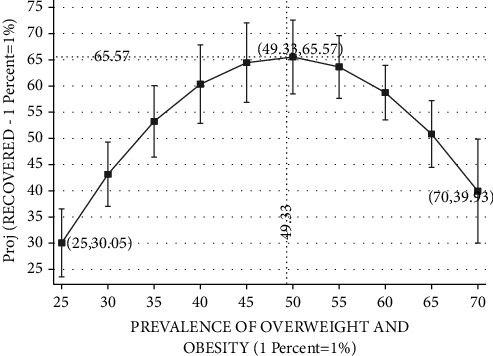
Rate of recovered COVID-19 patients vs. prevalence of overweight and obesity. The vertical axis measures the projected probability of recovery divided by total cases of COVID-19 in percentage points. The horizontal axis measures the prevalence of overweight and obesity (BMI ≥ 25, where BMI=kg/meter^2^) in the population of OECD countries based on measured height and weight. The figure is based on the following regression outcomes given in column (1).

**Figure 6:**
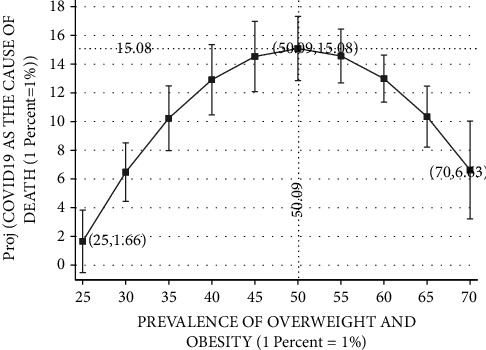
COVID-19 as the cause of death vs. prevalence of overweight and obesity. The vertical axis measures the projected probability of SARS-COV-2 virus as the cause of death rather than other reasons. The horizontal axis measures the prevalence of overweight and obesity (BMI ≥ 25, where BMI=kg/meter^2^) in the population of OECD countries based on measured height and weight. The figure is based on the following regression outcomes given in column (1).

**Figure 7:**
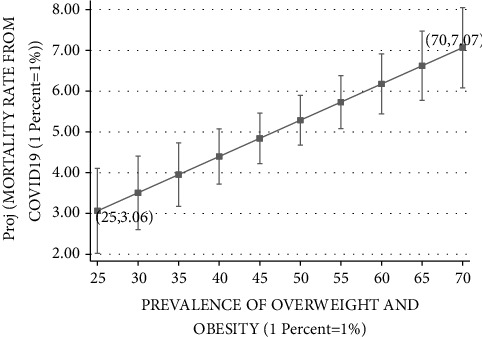
Mortality rate from COVID-19 vs. prevalence of overweight and obesity. The vertical axis measures the projected probability of mortality from COVID-19 based on the formula for each OECD country: 100n(NUMBER OF DEATHS FROM COVID19*÷*TOTAL NUMBER OF COVID19 PATIENTS). The horizontal axis measures the prevalence of overweight and obesity (BMI ≥ 25, where BMI=kg/meter^2^) in the population of 23 OECD countries based on measured height and weight. The figure is based on the following regression outcomes given in column (2).

Figures 3–7 plot the results based on the regression analysis given at the bottom of each figure. The vertical axis in these graphs includes the following SARS-CoV-2 virus indicators: projected rate of (1) infections by SARS-CoV-2 virus per 10,00,000 persons, (2) severe SARS-CoV-2 virus case, (3) recovered SARS-CoV-2 virus patients, (4) mortality from SARS-CoV-2 virus (rather than other reasons), and (5) mortality from SARS-CoV-2 virus of the total SARS-CoV-2 virus cases. The horizontal axis describes the prevalence of overweight and obesity within the range of 25 percent to 70 percent.

**Table:** 

Variables	(1)	(2)
	Infected	Infected
Overweight^2^	−2.269^∗∗∗^	—
	(0.00274)	—
Overweight	236.2^∗∗∗^	36.92^∗∗∗^
	(0.000457)	(3.51 × 10^−5^)
Constant	−4,202^∗∗∗^	−514.1
	(0.00134)	(0.251)
Observations	180	180
OECD Countries	23	23
R-squared	0.137	0.092

*P* values are given in parentheses. ^∗^*P* < 0.1; ^∗∗^*P* < 0.05; ^∗∗∗^*P* < 0.01. The Ramsey RESET test is based on estimating the model: $Y_{1}=a_{1}+b_{1}X+c_{1}{\hat{Y}}_{1}^{2}+\mu_{1}$, where ${\hat{Y}}_{1}={\hat{d}}_{1}+{\hat{e}}_{1}X$; *Y*1 = INFECTED; *X* = OVERWEIGHT; H0: *c*_1_=0; and H1: *c*_1_ ≠ 0. The test supports the hypothesis that the quadratic model better fits the data (calculated F(1,177) = 9.23; *P*=0.0027).

**Table:** 

Variables	(1)	(2)
	Severe	Severe
Overweight^2^	0.00204^∗∗∗^	—
	(<0.0001)	—
Overweight	−0.197^∗∗∗^	−0.0179^∗∗∗^
	(<0.0001)	(5.86 × 10^−8^)
Constant	5.437^∗∗∗^	2.122^∗∗∗^
	(<0.0001)	(<0.0001)
Observations	180	180
OECD Countries	23	23
R-squared	0.409	0.153

*P* values are given in parentheses. ^∗^*P* < 0.1; ^∗∗^*P* < 0.05; ^∗∗∗^*P* < 0.01. The Ramsey RESET test is based on estimating the model: $Y_{2}=a_{2}+b_{2}X+c_{2}{\hat{Y}}_{2}^{2}+\mu_{2}$, where ${\hat{Y}}_{2}={\hat{d}}_{2}+{\hat{e}}_{2}X$, *Y*2 = SEVERE, *X* = OVERWEIGHT, H0: *c*_2_=0, and H1: *c*_2_ ≠ 0. The test supports the hypothesis that the quadratic model better fits the data (calculated F(1,177) = 76.75; *P* < 0.0001; *P*=0.2238).

**Table:** 

Variables	(1)	(2)
	Recovered	Recovered
Overweight^2^	−0.0600^∗∗∗^	—
	(2.91 × 10^−8^)	—
Overweight	5.919^∗∗∗^	0.644^∗∗∗^
	(1.05 × 10^−9^)	(1.62 × 10^−9^)
Constant	−80.42^∗∗∗^	16.49^∗∗∗^
	(9.55 × 10^−6^)	(0.0114)
Observations	151	151
OECD Countries	22	22
R-squared	0.305	0.143

*P* values are given in parentheses. ^∗^*P* < 0.1; ^∗^*P* < 0.05; ^∗∗∗^*P* < 0.01. The Ramsey RESET test is based on estimating the model: $Y_{3}=a_{3}+b_{3}X+c_{3}{\hat{Y}}_{3}^{2}+\mu_{3}$, where ${\hat{Y}}_{3}={\hat{d}}_{3}+{\hat{e}}_{3}X$, *Y*3 = RECOVERED, *X* = OVERWEIGHT, H0: *c*_3_=0, and H1: *c*_3_ ≠ 0. The test supports the hypothesis that the quadratic model better fits the data (calculated F(1,148) = 34.32; *P* < 0.0001).

**Table:** 

Variables	(1)	(2)
	COVID-19	COVID-19
Overweight^2^	−0.0213^∗∗∗^	—
	(5.14 × 10^−9^)	—
Overweight	2.137^∗∗∗^	0.272^∗∗∗^
	(5.79 × 10^−11^)	(2.05 × 10^−9^)
Constant	−38.43^∗∗∗^	−4.199^∗∗∗^
	(7.72 × 10^−10^)	(0.0539)
Observations	165	165
OECD Countries	21	21
*R*-squared	0.351	0.198

*P* values are given in parentheses. ^∗^*P* < 0.1; ^∗∗^*P* < 0.05; ^∗∗∗^*P* < 0.01. The Ramsey RESET test is based on estimating the model: $Y_{4}=a_{4}+b_{4}X+c_{4}{\hat{Y}}_{4}^{2}+\mu_{4}$, where ${\hat{Y}}_{4}={\hat{d}}_{4}+{\hat{e}}_{4}X$, *Y*4=COVID-19, *X* = OVERWEIGHT, H0: *c*_4_=0, and H1: *c*_4_ ≠ 0. The test supports the hypothesis that quadratic model better fits the data (calculated F(1,162) = 38.13; *P*=0.2238).

**Table:** 

Variables	(1)	(2)
	Mortality	Mortality
Overweight^2^	−0.00196	—
	(0.224)	—
Overweight	0.261^∗^	0.0890^∗∗∗^
	(0.0678)	(2.45*e* − 06)
Constant	−2.340	0.839
	(0.399)	(0.372)
Observations	180	180
OECD Countries	23	23
R-squared	0.125	0.118

*P* values are given in parentheses. ^∗^*P* < 0.1; ^∗∗^*P* < 0.05; ^∗∗∗^*P* < 0.01. The Ramsey RESET test is based on estimating the model: $Y_{5}=a_{5}+b_{5}X+c_{5}{\hat{Y}}_{5}^{2}+\mu_{5}$, where ${\hat{Y}}_{5}={\hat{a}}_{5}+{\hat{b}}_{5}X$, *Y*5 = MORTALITY, *X* = OVERWEIGHT, H0: *c*_2_=0, and H1: *c*_2_ ≠ 0. The test supports the hypothesis that the linear model better fits the data (calculated F(1,177) = 1.49; *P*=0.2238).

Figure 3 reports the relationships between SARS-CoV-2 virus cases per 10,00,000 persons and the prevalence of overweight and obesity. The Ramsey RESET test supports the quadratic in favor of the linear model (calculated F(1,177) = 90.23; *P*=0.0027). Projected SARS-CoV-2 virus cases begin with approximately 285.9 per 10,00,000 persons in a country, such as Japan, which exhibits the lowest obesity prevalence of 25 percent. Projected SARS-CoV-2 virus cases steadily rise with the prevalence of obesity until peaking at approximately 1,947.2 per 10,00,000 persons in countries such as Israel and the Czech Republic, which exhibit overweight and obesity prevalence of 52.05 percent. At this range of 25–52.05 prevalence of overweight and obesity, upward changes seem to exacerbate the situation and increase the number of SARS-CoV-2 virus infected per 10,00,000 persons. Yet, referring to an upward change above obesity prevalence of 52.05 percent, this SARS-CoV-2 virus indicator has a beneficial effect, dropping to approximately 1,217 per 10,00,000 persons for 70 percent obesity prevalence, in countries such as the United States, Mexico, and Chile.

Figure 4 reports the relationships between the rate of severe SARS-CoV-2 virus cases of the total number of SARS-CoV-2 virus cases and the prevalence of overweight and obesity. The Ramsey RESET test supports the quadratic model in favor of the linear model (calculated F(1,177) = 76.75; *P* < 0.0001). Projected rate of severe SARS-CoV-2 virus cases begins with 1.78 percent in a country, such as Japan. Projected rate of severe SARS-CoV-2 virus cases steadily drops with the prevalence of obesity until it reaches its nadir with 0.67 percent of severe cases in countries such as Israel and the Czech Republic, which exhibit overweight and obesity prevalence of 48.32 percent. At this range of 25–48.32 prevalence of overweight and obesity, upward changes seem to have a beneficial effect. Yet, referring to an upward change above obesity prevalence of 48.32 percent, this SARS-CoV-2 virus indicator has a worsening effect. It rises to approximately 1.63 percent of severe SARS-CoV-2 virus cases, in countries such as the United States, Mexico, and Chile.

Figure 5 reports the relationships between the rate of recovered SARS-CoV-2 virus patients and the prevalence of overweight and obesity. Again, the Ramsey RESET test supports the quadratic model in favor of the linear model (calculated F(1,148) = 34.32; *P* < 0.0001). Our findings suggest a beneficial effect in the 25–49.33 percent prevalence of obesity range with an elevated prevalence of obesity and a worsening effect within the 49.33–70 percent range. Projected rate of recovered SARS-CoV-2 virus patients starts with approximately 30.05 percent in a country, such as Japan. The projected rate of recovered SARS-CoV-2 virus patients steadily rises with the prevalence of obesity, peaking at approximately 65.57 percent in countries such as Israel and the Czech Republic, which exhibit overweight and obesity prevalence of 49.33 percent. Referring to an upward change above obesity prevalence of 49.33 percent, this SARS-CoV-2 virus indicator drops to approximately 39.93 recovery rate for 70 percent obesity prevalence, in countries such as the United States, Mexico, and Chile.

Figure 6 reports the relationships between SARS-CoV-2 virus as the cause of death of the total number of dead persons and the prevalence of overweight and obesity. As noted previously, the Ramsey RESET test supports the quadratic model in favor of the linear model (calculated F(1,162) = 38.13; *P* < 0.0001). Projected SARS-CoV-2 virus cases begin with approximately 1.66 percent in Japan, the OECD country with the lowest obesity prevalence. Projected rate of SARS-CoV-2 virus as the cause of death steadily rises with the prevalence of obesity until it reaches its peak of approximately 15.08 percent in countries such as Israel and the Czech Republic, which exhibit overweight and obesity prevalence of 50.09 percent. At this range of 25–50.09 prevalence of overweight and obesity, upward changes seem to inflate SARS-CoV-2 virus as the cause of death. Yet, referring to an upward change above obesity prevalence of 50.09 percent, the frequency of SARS-CoV-2 virus as the reason of death drops to approximately 6.63 percent for 70 percent obesity prevalence, in the United States, Mexico, and Chile.

Finally, Figure 7 reports the relationships between mortality rate from SARS-CoV-2 virus and the prevalence of overweight and obesity. Consistent with the previous analyses, the Ramsey RESET test supports the linear model in favor of the quadratic model (calculated F(1,177) = 1.49; *p*=0.2238). For the lowest obesity prevalence of 25 percent, the projected mortality rate is 3.06. This projected SARS-CoV-2 virus indicator steadily rises with the prevalence of obesity until it reaches a peak of the projected mortality rate of 7.07 percent for 70 percent obesity prevalence, in countries such as the United States, Mexico, and Chile.

## 5. Discussion

In this section, we compare the differences and similarities between the current manuscript applied to OECD countries, and Arbel et al. [17] applied to the US states.

USA is a member of the OECD with a high prevalence of overweight and obesity (third place after Mexico and Chile with 70% of the population considered to be overweight, namely, BMI ≥ 25). The dates of studies are quite similar: in US states May 5, 2020, and in OECD countries April 28, 2020..

Referring to the OECD countries, the information considered is the prevalence of overweight (BMI ≥ 25), whereas in the study of US states, the independent variable is the prevalence of obesity (BMI ≥ 30) in US states. Unlike the US research, the contribution of the OECD study emanates from additional indicators apart from the rate of COVID-19 morbidity and mortality. These include the rate of severe cases, and the percent of COVID-19 of the total mortality.

In contrast to the US research, which focused on one country, the current study exhibits more extensive global perspective. OECD countries span four different continents: Asia, Europe, Australia, and America (North, Central, and South).

With respect to the scope of morbidity measured as COVID-19 cases per 1 million persons, the difference is as follows. While in research conducted regarding US states, the anticipated scope of morbidity declines monotonically from 6,200 cases per million persons in states with 20% prevalence of obesity to fewer than 1,700 cases per million persons in states with 40% prevalence of obesity, in OECD countries, the decline is not monotonic for the full range. Accordingly, starting from 52% prevalence of overweight, there is a decline from a maximum of 1,947 to 1,217 COVID-19 cases per 1 million persons in countries with 70% prevalence of overweight. One possible explanation is cultural differences and concurrent differences in eating habits, among residents in the various OECD countries.

Referring to the scope of COVID-19 mortality, the trend is reversed. While in US states, there is a monotonic decline with a higher prevalence of obesity starting from an anticipated 5.35 percent (20% prevalence of obesity) to less than 3.46 percent (40% prevalence of obesity), in OECD countries, there is a monotonic increase from an anticipated 3.06 percent (25% prevalence of overweight) to 7.07 percent (70% prevalence of overweight). A potential explanation is political regime differences in OECD countries. As of 2021, the OECD consists of 38 members, of which, on a 2020 scale of 1 = the best, 7 = the worst ranking in terms of political rights, 30 members are defined as “one,” 4 members are defined as “two,” 3 members are defined as “3”(Columbia, Hungary, and Mexico), and one country is defined as “5” (Turkey).

As the relevant literature demonstrates, in more democratic countries, the tendency to invest in health infrastructure is higher, in light of the need for reelection, e.g., [18].

## 6. Conclusion

The objective of the current study is to analyze the potential relationships between different SARS-CoV-2 virus indicators and obesity on a country-wide level, using the OECD countries as the basis for the comparison. The dependent variables in our model are the following: (1) infections by SARS-CoV-2 virus per 10,00,000 persons, (2) rate of severe SARS-CoV-2 virus case, (3) rate of recovered SARS-CoV-2 virus patients, (4) rate of mortality from SARS-CoV-2 virus (rather than other reasons), and (5) mortality from SARS-CoV-2 virus of the total SARS-CoV-2 virus cases. The independent variable includes overweight and obesity prevalence on a scale of between 25 percent (Japan) and approximately 70 percent (USA, Mexico, and Chile). Given the counterintuitive possibility of an obesity survival paradox [5, 19–21], the current study relaxes the assumption of monotonic change by applying the quadratic design and testing which of the two competing models (i. e., quadratic or linear) better fits the data.

Findings suggest that relationships between SARS-CoV-2 virus indices and obesity rates are more complex than previously thought. Consequently, ethical guidelines referring to priority in intubation and intensive care treatments—published by the Israeli Ministry of Health in April 2020—should account for these complex relationships between obesity and SARS-CoV-2 virus. Indeed, there is a linear increase in mortality rate from SARS-CoV-2 virus with the prevalence of obesity. Nevertheless, other indicators, such as the number of infected per 10,00,000 persons from the population, rates of severe SARS-CoV-2 virus cases, rates of recovered SARS-CoV-2 virus patients, and SARS-CoV-2 virus, as the cause of death exhibit quadratic rather than linear patterns. Given the (1) decrease in the number of infected per 10,00,000 persons for overweight and obesity prevalence of 50–70%, (2) drop in the rate of severe cases for overweight and obesity prevalence of 25–50%, and (3) increase in the recovery rates for overweight and obesity prevalence of 25–50%, these patterns give rise to the possibility of the obesity survival paradox.

Referring to pneumonia and different types of cancer, including melanoma, the literature identifies the potential existence of the obesity survival paradox, namely, the better prospects of obese persons to recover and lower prospects to be infected, e.g., [19–22]. This may be explained as follows:

1. Social and Behavioral Aspects of the Individual: the lower inclination of obese persons to engage in physical activity and walks outside the home (e.g., [23–26], which leads, in turn, to reduced interactions with other persons.
2. Behavioral Patterns in the Health System: more aggressive treatment regimens following the conventional definition of obesity as a global pandemic and a risk factor for a long series of health problems, including elevated mortality risk (e.g., [22, 27, 28].
3. Physiological Considerations: metabolic reserves of obese persons, which may protect against mortality (e.g., [20]).

Several limitations of this research may be pointed out. This study is a cross-sectional description of OECD countries, which was prepared at an earlier stage of the COVID-19 pandemic. Examining the evolvement of the pandemic in OECD countries is a subject for future research upon arrival of new data. On the one hand, the use of data from April 2020 has a significant advantage, namely, the period where the only variant was the delta variant. On the other hand, since the current research cannot address the developments of new variants, vaccinations, etc., this may be considered part of the limitations of the research. Also, the comparison is between OECD countries, which enacted different intervention policies. While Brazil has experienced peak levels in some states today, in Israel, the lockdowns and massive vaccination operation were successful in reducing the pandemic. Finally, the model lacks confounders, such as population heterogeneity within the various countries.

## Figures and Tables

**Table 1:** Definition of indicators for COVID-19 and obesity.

Variable	Formula	Definition
Infected	1,000,000 · [COVID19_CASES/POPULATION]	Number of COVID-19 cases per 10,00,000 persons.
Severe	100 · [SEVERE_CASES/COVID19_CASES]	Relative prevalence of severe COVID-19 cases (1 percent = 1%).
Recovered	100 · [RECOVERY_CASES/COVID19_CASES]	Relative prevalence of recovery (1 percent = 1%).
COVID-19	Already defined in % (1 percent = 1%)	COVID-19 as the cause of death from the total number of dead persons (1 percent = 1%).
Mortality	100 · [DEATH_CASES/COVID19_CASES]	Relative frequency of death from COVID-19 (1 percent = 1%).
Overweight	Already defined in %	Relative frequency (1 percent = 1%) of overweight in the country (BMI ≥ 25).
Year	Irrelevant	The year in which the measurement took place.

**Table 2:** Descriptive statistics stratified by 23 OECD countries.

Countries	Population	INFECTED	SEVERE	RECOVERED	COVID-19	MORTALITY	OVERWEIGHT
	Population of the country	COVID-19 cases per 10,00,000 persons	Relative prevalence of severe COVID-19 cases (1 percent = 1%)	Relative prevalence of recovery (1 percent = 1%)	COVID-19 as the cause of death from the total number of dead persons (1 percent = 1%).	Relative frequency of death from COVID-19 (1 percent = 1%).	Relative frequency (1 percent = 1%) of overweight in the country (BMI ≥ 25).
AUS	25,203,198	267	0.62	84	0.5	1.25	54.05
BEL	11,539,328	4,102	1.85	23	43	15.49	51.00
CAN	37,411,047	1,296	1.15	38	3.5	5.58	59.51
CHL	18,952,038	729	3.08	53	0.9	1.43	67.00
CZE	10,689,209	697	0.98	38	1.5	2.99	52.00
DEU	83,517,045	1,901	1.52	74	4	3.86	59.85
EST	1,325,648	1,252	0.54	14	2.2	3.01	51.30
FIN	5,532,156	857	1.18	53	1.3	4.07	61.56
FRA	65,129,728	2,546	2.78	27	30	14.05	49.00
GBR	675,301,372	233	0.99	—	23	13.42	57.73
HUN	9,684,679	274	1.85	19	1	10.99	56.53
IRL	4,882,495	4,024	0.74	47	15	5.61	61.33
ISR	8,519,377	1,830	0.75	47	3.1	1.33	56.55
JPN	126,860,301	107	2.20	14	0.1	2.83	22.76
KOR	51,225,308	210	0.51	82	0.9	2.27	30.89
LUX	615,729	6,056	0.54	84	23	2.36	54.10
LVA	1,906,743	438	0.48	32	2.2	1.56	54.60
MEX	127,575,529	122	2.43	59	0.5	9.23	67.94
NZL	4,783,063	308	0.07	82	—	1.29	62.20
PRT	1,906,743	12,756	0.71	6	5.8	3.90	67.60
SVK	5,457,013	254	0.43	31	—	1.45	51.07
TUR	83,429,615	1,346	1.55	30	3.7	2.58	59.90
USA	329,064,917	3,071	1.40	14	10	5.62	64.82

*Note*: The table reports the sample mean for 23 OECD countries. Information on the frequency of overweight in different countries is available at: https://data.oecd.org/pinboard-editor/ (Last accessed on June 30, 2021). Information regarding the COVID-19 is updated till April 28, 2020. The population data are updated till July 2019.

**Table 3:** Descriptive statistics–pooled sample.

Variable	N	OECD countries	Mean	Median	(Standard error)
Infected	180	23	1,275.96^∗∗∗^ [973.82, 1,578.09] {877.32, 1,674.59}	243.16	(153.11)
Severe	180	23	1.25^∗∗∗^ [1.14, 1.37] {1.10, 1.40}	0.99	(0.0577)
Recovered	151	22	46.57^∗∗∗^ [41.75, 51.40] {40.20, 52.95}	38.15	(2.44)
COVID-19	165	21	8.73^∗∗∗^ [7.12, 10.34] {6.60, 10.85}	1.5	(0.815)
Mortality	180	23	5.15^∗∗∗^ [4.51, 5.80] {4.30, 6.00}	2.83	(0.33)
Overweight	180	23	48.49^∗∗∗^ [46.01, 50.97] {45.22, 51.76}	54.6	(1.26)
Year	180	23	2004.26 [2002.75, 2005.76] {6.60, 10.85}	2007	(0.76)

*Note*: The YEAR variable spans from 1978 to 2018. The OVERWEIGHT variable is calculated as the prevalence of persons in the population whose BMI ≥ 25(kg/meter^2^). Standard errors = $\text{Standard Deviations}/\sqrt{N}$ are given in parentheses. [95%] {99%} confidence intervals are given in [square] {curly} brackets. ^∗∗∗^ for rejection of the null hypothesis of equality to zero.

## Data Availability

The data used to support the findings of this study, obtained from the OECD website, are available from the corresponding author upon request.
